# Effects of Trehalose Solutions at Different Concentrations on High-Intensity Intermittent Exercise Performance

**DOI:** 10.3390/nu14091776

**Published:** 2022-04-23

**Authors:** Naomi Hamada, Tsuyoshi Wadazumi, Yoko Hirata, Hitoshi Watanabe, Nobuko Hongu, Norie Arai

**Affiliations:** 1 Graduate School of Health and Well-Being, Department of Health and Well-Being, Kansai University, 1-11-1, Kaorigaoka-cho, Sakai-ku, Sakai 590-8515, Osaka, Japan; wadazumi@kansai-u.ac.jp (T.W.); yo-hirata@kwjc.kobe-wu.ac.jp (Y.H.); 2 Department of Applied Food Science, Higashiosaka Junior College, 3-1-1, Nishizutsumigakuen-cho, Higashiosaka 577-8567, Osaka, Japan; 3 Department of Food and Nutritional Science, Kobe Women’s Junior College, 4-7-2, Nakamachi, Minatojima, Chuo-ku, Kobe 650-0046, Hyogo, Japan; 4 Research Center for Urban Health and Sports, Osaka Metropolitan University, 3-3-138, Sugimoto, Sumiyoshi-ku 558-8585, Osaka, Japan; ocuwatanabe@gmail.com; 5 Graduate School of Human Life Science, Department of Food and Human Life Science, Osaka Metropolitan University, 3-3-138, Sugimoto, Sumiyoshi-ku 558-8585, Osaka, Japan; kay.hongu@gmail.com; 6 Hayashibara, Co., Ltd., 675-1, Fujisaki, Naka-ku 702-8006, Okayama, Japan; norie.arai@hb.nagase.co.jp

**Keywords:** trehalose, concentration, exercise performance, blood glucose

## Abstract

Trehalose solution ingested during exercise induces gradual increases in blood glucose levels and the insulin response compared with glucose solution. Trehalose solution aids in the maintenance of performance in the later stages of prolonged exercise. The purpose of this study was to identify the lowest concentration at which the properties of trehalose could be exploited. Groups of 12 healthy men (21.3 ± 1.3 years) and 10 healthy men (21.1 ± 0.7 years) with recreational training were included in experiments 1 and 2, respectively. Both experiments followed the same protocol. After fasting for 12 h, the participants performed a 60 min constant-load exercise at 40% $\dot{V}$O_2_ peak using a bicycle ergometer and ingested 500 mL of a trial drink (experiment 1: water, 8% glucose, and 6 or 8% trehalose; experiment 2: 4 or 6% trehalose). They performed four sets of the Wingate test combined with a 30 min constant-load exercise at 40% $\dot{V}$O_2_ peak. The experiment was conducted using a randomized cross-over design; trial drink experiments were conducted over intervals of 7 to 12 days. The exercise performance was evaluated based on mean power in the Wingate test. Blood was collected from the fingertip at 12 points during each experiment to measure blood glucose levels. During the high-intensity 5 h intermittent exercise, no differences were found between the groups in exercise performance in the later stages with concentrations of 8, 6, and 4% trehalose solution. The results suggest that trehalose could be useful for making a new type of mixed carbohydrate solution. Further studies to determine the trehalose response of individual athletes during endurance exercise are needed.

## 1. Introduction

Carbohydrates and lipids are the primary energy sources for endurance exercise [1]. Since the body stores lower amounts of carbohydrates than lipids, carbohydrates consumed in the diet are stored as glycogen in the skeletal muscle and liver and used as an energy source during exercise [2]. The stored glycogen is highly likely to be depleted during prolonged moderate- to high-intensity exercise. Therefore, carbohydrate supplementation is needed for exercise to continue [3]. It is well established that ingesting carbohydrates during endurance exercise substantially improves performance [4,5,6,7], and it is recommended to ingest 30–60 g/h of glucose during exercise according to the rate of blood glucose consumption [8,9,10]. Endurance exercise performance increases depending on carbohydrate intake during exercise, but the effect generally plateaus at a dose of 60 g/h (1.0 g/min) [8,11]. This is probably because ingesting more than 60 g/h of glucose alone saturates the capacity of sodium-glucose cotransporter 1 (SGLT1), which mediates glucose absorption in the small intestine, and carbohydrate absorption reaches its limit [12]. However, ingesting a mixture of multiple types of carbohydrates, such as glucose and fructose, increases the rate of blood glucose absorption to 90 g/h (1.5 g/min) through the involvement of their respective transporters, resulting in improved endurance exercise performance compared with the ingestion of glucose alone [13]. Recently, hypotonic beverages using highly branched cyclic dextrin (HBCD) have been developed. HBCD is set to a low osmotic pressure, which can shorten the residence time in the stomach [14]. As described above, the use of mixed carbohydrate solutions taking advantage of the absorption characteristics of each carbohydrate (glucose, maltose, oligosaccharides, trehalose, etc.) is being promoted in the field of sports nutrition as a new approach that has not been used in conventional carbohydrate supplementation [15,16].

Naturally occurring carbohydrates include trehalose (α-D-glucopyranosyl-α-D-glucopyranoside), a disaccharide composed of two glucose molecules linked by an α,α-1,1-glycosidic linkage [17,18]. Trehalose is present in common foods such as mushrooms, yeast, and seaweed [19]. It is extracted from foods in small amounts and is considered a rare sugar. However, the establishment of an enzymatic method for producing trehalose has enabled its mass production at low cost. Trehalose has been increasingly used in the food industry in various ways [20,21].

A previous study by Wadazumi et al. [22] compared the performance benefits of single ingestion of 8% trehalose solution and 8% glucose solution during the later stages of prolonged exercise. They reported that the trehalose solution caused a less rapid increase in blood glucose and insulin levels than glucose solution and maintained performance until the later stage of exercise. In another study [23], blood biochemical analysis was performed using the same experimental methods (the Wadazumi protocol). The results showed that 8% trehalose solution successfully suppressed the rapid increase in insulin and blood glucose compared to 8% glucose solution. This allows the body to maintain the glucose necessary for performance in the later stages of exercise without decreasing the exercise efficiency. Carbohydrates in the body are preserved, which means that carbohydrate waste is suppressed.

Trehalose has the property of being absorbed more slowly than glucose due to the enzymatic effects of trehalase [17]. In addition, the sweetness of trehalose is approximately 38% that of sucrose [24]. Trehalose has some advantage when it is prepared in a mixed solution with other carbohydrates. Trehalose has limited effects on taste (i.e., it is not too sweet); therefore, combining it with other carbohydrates may make it easier for some athletes to consume it. Lastly, an increased concentration of carbohydrates in the mixed solution can reduce the gastric emptying rate, which can cause abdominal discomfort or affect exercise performance in some athletes. Therefore, the concentration of mixed carbohydrate solution may need to be 8% or less [25,26].

Thus, in this study, we aimed to determine the lowest single-dose trehalose concentration required to maintain high performance during prolonged exercise that can take advantage of the characteristics of trehalose. Normally, low-carbohydrate concentrations result in energy deficiency. However, we hypothesized that the body could take advantage of the carbohydrate-saving properties of trehalose, even at low concentrations.

In experiment 1, the effects of carbohydrate solution on exercise performance were examined using a 6% trehalose solution, with an 8% glucose solution as control, as well as an 8% trehalose solution known to have an effect on maintaining and improving performance in the later stages of exercise. In experiment 2, an even lower trehalose concentration of 4% was selected and compared to the effects of 6% trehalose solution.

## 2. Materials and Methods

### 2.1. Participants

All participants received full information regarding the objectives, experimental methods, and possible risks of the study. Written informed consent was obtained from those who voluntarily agreed to participate in the study.

The participants were instructed to maintain their normal diet and physical activity during the experimental period. We did not strictly define their daily caloric intake or ask them to record the amount of energy or micronutrients in their diet. In addition, they were not allowed to perform vigorous physical activity, ingest caffeine, or drink alcohol 24 h before each trial drink experiment. They were allowed to drink water but fasted from 21:00 on the day before each trial drink experiment to make it easier to produce the state of depleted muscle glycogen. Since the time and quality of sleep would have a great influence, we instructed them to have enough sleep the day before the experiment. The experiment was conducted after investigating the participants’ condition during the experiment and confirming that they had no sleep problems. Exclusion criteria were known heart disease, diabetes, or any disorder that prohibited participation in the testing procedures.

#### 2.1.1. Experiment 1

Experiment 1 was conducted with 12 healthy male college students (age 20–24 years) with a habit of at least 180 min of regular exercise each week, who volunteered to participate (Table 1). 

#### 2.1.2. Experiment 2

Experiment 2 was conducted with 10 healthy male college students (age 20–22 years) who were not enrolled in experiment 1. They had a habit of at least 180 min of regular exercise each week (Table 1). The procedure of experiment 2 was the same as experiment 1, except for the type of trial drink (6% and 4% trehalose solution) ingested after the first set of the Wingate test. 

### 2.2. Ethics

In accordance with the Declaration of Helsinki, this study was conducted with ethical consideration of the human rights and personal information of the participants. Ethical approval for this study was obtained from the Ethics Committee of the Faculty of Health and Well-Being, Kansai University (approval no. 2018-16). This study was registered with the University Hospital Medical Information Network Center (UMIN study no. UMIN000046293). All hard-copy study data were stored in a locked filing cabinet. Electronic data were stored on a secure network drive, with access granted only to those working in the laboratory.

### 2.3. Trial Drinks

Glucose (FUJIFILM Wako Pure Chemical Corporation, Osaka, Japan) and trehalose (TRAHA^®^; Hayashibara Co., Ltd., Okayama, Japan) were used as carbohydrates.

#### 2.3.1. Experiment 1: 8% Glucose Solution, 8% Trehalose Solution, 6% Trehalose Solution, Water

The 8% glucose solution (G8) and 8% trehalose solution (T8) were prepared by dissolving 40 g of trehalose or glucose in water to obtain a total volume of 500 mL. The 6% trehalose solution (T6) was prepared by dissolving 30 g of trehalose in water to obtain a total volume of 500 mL of water (W), which was used as a control.

#### 2.3.2. Experiment 2: 6% and 4% Trehalose Solution

The 6% trehalose solution (T6) and 4% trehalose solution (T4) used as trial drinks were prepared by dissolving 30 and 20 g of trehalose, respectively, in water to obtain a total volume of 500 mL.

### 2.4. Preliminary Testing

At least 1 week before the start of the experiment, preliminary testing of all participants was performed using an electromagnetic brake-type bicycle ergometer (75XLIII aero bike, Konami Holdings Corporation, Tokyo, Japan) and an expired gas analyzer (AE-310S aero monitor, Minato Medical Science Co., Ltd., Osaka, Japan). Based on the detected values, the exercise load (in watts) corresponding to a 40% $\dot{V}$O_2_ peak was determined for each participant. Additionally, the participants practiced the Wingate test (PowerMax III, Konami Holdings Corporation, Tokyo, Japan) in advance and performed the main experiments after becoming familiar with the test.

### 2.5. Experimental Design

The participants came to the laboratory at 09:00 a.m. for body composition measurements. The body weight and body fat percentage were measured to the nearest 0.5 kg with the participants in light training gear and without shoes, using a balance beam scale (HBF-214; Omron Corporation, Kyoto, Japan). Height was measured to the nearest 0.1 cm with a wall-mounted stadiometer (UY-2, Uchida Yoko Co., Ltd., Tokyo, Japan). Subsequently, experiment 1 was started in an experimental room maintained at a temperature of 25 °C and humidity of 50%. The experiment was conducted using a randomized, double-blind cross-over design. All participants performed the experiments based on the same protocol, ingesting different carbohydrate drinks: 8% glucose solution, 6 or 8% trehalose solution, or water (control). Each experiment consisted of approximately 5 h of cycling combining constant-load exercise at 40% $\dot{V}$O_2_ peak with ultra-high-intensity intermittent exercise (Wingate test) according to the Wadazumi protocol, described in the following section. All participants performed the trial drink experiments at least 1 week (7 days) apart. However, in the case of poor physical condition, an extension of up to 5 days was allowed, resulting in an interval of 7 to 12 days.

#### 2.5.1. Wadazumi Protocol

**Figure 1:**
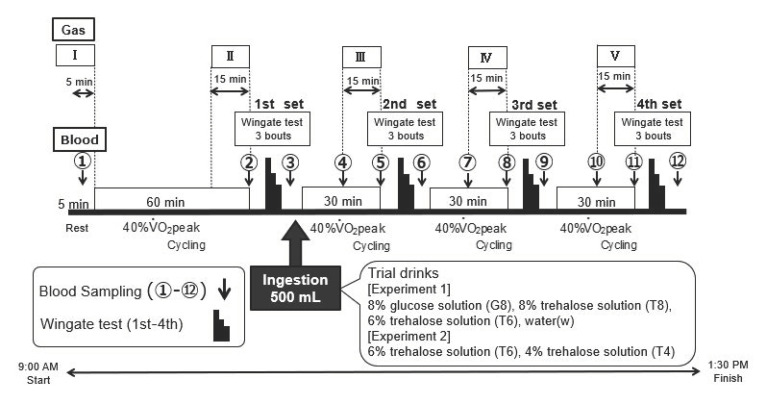
Experimental design (Wadazumi protocol). This cycling exercise protocol combines constant-load exercise and the Wingate test. Intensity of constant load was set at 40% $\dot{V}$O_2_ peak for 60 min and 30 min × 3 times. Four sets of the Wingate test (30 s × 3 times) were performed. After the first Wingate test, participants ingested a trial drink. Expired gas was collected at rest (I) and in last 15 min of 60 and 30 min constant-load exercise (II–V). Points ① to ⑫ indicate blood sample collection, RPE measurements, and Borg scale measurements.

**Figure 2:**
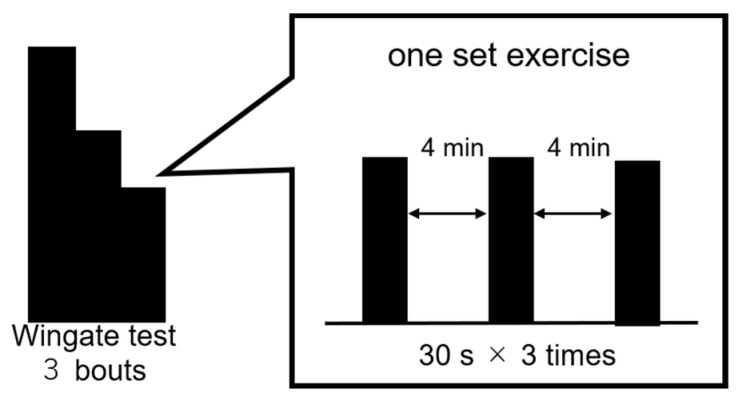
Wingate test.

The experimental protocol comprised a combination of constant-load exercise and the Wingate test (Figure 1 and Figure 2) [21]. The participants performed constant-load exercises at 40% $\dot{V}$O_2_ peak for 60 min using an electromagnetic brake-type bicycle ergometer. Subsequently, they performed 4 sets of the Wingate test in combination with constant-load exercise at 40% $\dot{V}$O_2_ peak. The constant-load exercise was performed for 30 min after the first, second, or third set of the Wingate test.

Within 5 min after the end of the first set of the Wingate test, the participants ingested 500 mL of a trial drink (see below) and were observed for their performance on the next 3 sets of the test. They were allowed free access to drink water, other than the trial drink, and the amount of water intake was recorded. Expired gas was collected at rest (I) and during the last 15 min of the 60 min or 30 min constant-load exercise (II–V).

#### 2.5.2. Wingate Test

Exercise performance was assessed using the Wingate test with an electromagnetic brake-type bicycle ergometer. Figure 2 shows the exercise mode. The pedals were subjected to a load of 0.075 kg/kg body weight. Each set of exercises consisted of 3 bouts of the 30 s Wingate test, with a 4 min recovery period between each bout. The investigator instructed the participants to continue full-power cycling during each exercise in all sets and encouraged them to exert their full power. Hereafter, the third session of the Wingate test was referred to as the third set (3rd) and the fourth session as the fourth set (4th).

### 2.6. Measurements

The measurement methods for experiments 1 and 2 are shown below.

#### 2.6.1. Exercise Performance

The Wingate test detects the average power value for the 30 s bout and the maximum power among all power values for the 5 s sub-period. In this study, the average power value was used for evaluation because it reflects the contribution rate of glycolysis as a basic index of exercise performance. The percentage ratio of the mean for the three average power values (in watts) of the second, third, or fourth set to that of the first set was calculated for each participant. 

#### 2.6.2. Blood Glucose Measurements

At 12 time points from immediately before the start of the experiment (at rest) to the end of the experiment (①–⑫ in Figure 1), blood was collected from a fingertip with a puncture device (Gentle; Sanwa Kagaku Kenkyusho Co. Ltd., Aichi, Japan) to measure blood glucose levels using a self-monitoring glucometer (Glutest Neo Alpha GT-1830; Sanwa Kagaku Kenkyusho Co. Ltd., Aichi, Japan). Blood sampling was performed during the cycling exercise at time points ➃, ⑦, and ⑩. Blood was collected with the participant in a seated position after dismounting the bicycle at the remaining time points. To assess the changes in blood glucose levels in the later stages of the exercise, the area under the curve (AUC) from time points ⑩ to ⑫ (hereafter, “AUC ⑩–⑫”) was calculated.

#### 2.6.3. Expired Gas Analysis

To determine the use of energy substrates, expired gas was collected (I–V in Figure 1) using an expired gas analyzer (Aero Monitor AE-310S; Minato Medical Science Co., Ltd., Osaka, Japan) to measure the respiratory exchange ratio (RER).

#### 2.6.4. Rating of Perceived Exertion (RPE)

At the same time as blood sampling, rating of perceived exertion (RPE) was determined using the Borg scale [27].

### 2.7. Statistical Analysis

All results obtained in this study are expressed as mean ± standard deviation (SD). The data were tested for normality before statistical testing using the Shapiro–Wilk test. All statistical tests were performed at a significance level of <0.05, using SPSS Statistics ver. 27.0 statistical analysis software (IBM Corporation, New York, USA).

In experiment 1, repeated-measures one-way analysis of variance (ANOVA) was performed. Mauchly’s sphericity test was used to assess the assumption of sphericity in advance, and if the assumption was violated, the degrees of freedom were adjusted using the Greenhouse–Geisser epsilon. Bonferroni’s multiple comparison test was used as a post hoc test for comparisons among trial drinks. Additionally, effect sizes (Cohen’s *d*) were calculated and classified as follows: small, 0.20 < *d* < 0.49; medium, 0.50 < *d* < 0.79; and large, 0.80 < *d*) [28].

In experiment 2, a paired *t*-test was used to determine the significance of differences between the two trial drinks. Effect sizes (*d*) were also calculated as in experiment 1.

## 3. Results

### 3.1. Mean Power Value

#### 3.1.1. Experiment 1

Table 2 shows the mean power values in the third and fourth sets of the Wingate test by trial drink (W, G8, T8, or T6). The mean power values decreased as the set proceeded with all trial drinks. Significant main effects of the trial drinks were found in the third (F [3, 33] = 6.601, *p* < 0.001, $\eta_{p}^{2}$ = 0.375) and fourth (F[3, 33] = 7.687, *p* < 0.001, $\eta_{p}^{2}$ = 0.411) sets in the later stages of exercise. In the fourth set, the mean power value was significantly higher for T8 (93.3 ± 5.8%) than G8 (88.9 ± 7.2%) (*p* < 0.05, *d* = 0.68), thus reproducing the results of the previous study [22]. Compared with W, the mean power value was significantly higher for T6 in the third set (*p* < 0.05, *d* = 1.55) and for T8 (*p* < 0.05, *d* = 1.12) and T6 (*p* < 0.05, *d* = 1.26) in the fourth set. No significant differences in mean power values were observed between T8 and T6 in the third or fourth set.

#### 3.1.2. Experiment 2

Table 3 shows the mean power values in the third and fourth sets of the Wingate test by trial drink (T6 and T4). No differences in mean power values were noted between trial drinks in the third or fourth set during the later stages of exercise.

### 3.2. Blood Glucose Levels

#### 3.2.1. Experiment 1

**Figure 3:**
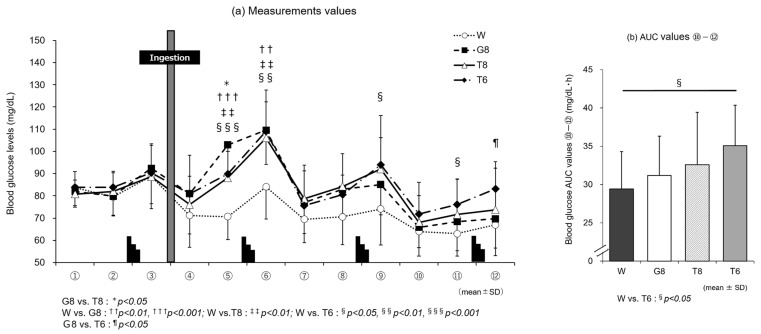
Changes in blood glucose levels with trial drinks (W, G8, T8, T6) (experiment 1). Comparison between (**a**) measurements and (**b**) area under the curve (AUC) ⑩–⑫ values of trial drinks (*n* = 12). G8 vs. T8: * *p* < 0.05; W vs. G8: ^††^ *p* < 0.01, ^†††^ *p* < 0.001; W vs. T8:^‡‡^ *p* < 0.01; W vs. T6: ^§^ *p* < 0.05, ^§§^ *p* < 0.01, ^§§§^ *p* < 0.001; G8 vs. T6: ^¶^ *p* < 0.05. W, water; G8, 8% glucose; T8, 8% trehalose; T6, 6% trehalose.

Figure 3a shows the changes in blood glucose levels by trial drink (W, G8, T8, and T6). The blood glucose levels increased after the end of each set of the Wingate test (time points ③, ⑥, ⑨, and ⑫) with all trial drinks, and no significant differences in blood glucose levels were found among the four trial drinks 15 min after ingestion (④). However, at 30 min after drink ingestion (⑤), the blood glucose level with W decreased slightly (70.7 + 10.3 mg/dL), and the blood glucose levels were significantly higher with all carbohydrate drinks (T8: 88.1 ± 14.9 mg/dL, *p* < 0.01; T6: 89.8 ± 10.2 mg/dL, *p* < 0.001; G8: 102.9 ± 16.4 mg/dL, *p* < 0.001) than with W. Notably, at time point ⑤ after carbohydrate ingestion, the increase was rapid for G8 and gradual for T8 and T6; the blood glucose level was significantly higher with G8 than T8 (*p* < 0.05, *d* = 0.95). However, after time point ⑦ in the later stages of exercise, the blood glucose levels remained higher for T8 and T6 than G8. At time point ⑫, the blood glucose level was significantly higher with T6 than G8 (*p* < 0.05, *d* = 1.05). A comparison of AUC values ⑩–⑫ in the later stages of exercise among the trial drinks (Figure 3b) revealed a significant main effect (F[3, 33] = 3.717, *p* < 0.05, $\eta_{p}^{2}$ = 0.253). Additionally, the AUC ⑩–⑫ was significantly higher with T6 (35.1 ± 5.3 mg/dL·h) than W (29.4 ± 4.9 mg/dL·h) (*p* < 0.05, *d* = 1.11).

##### 3.2.2. Experiment 2

**Figure 4:**
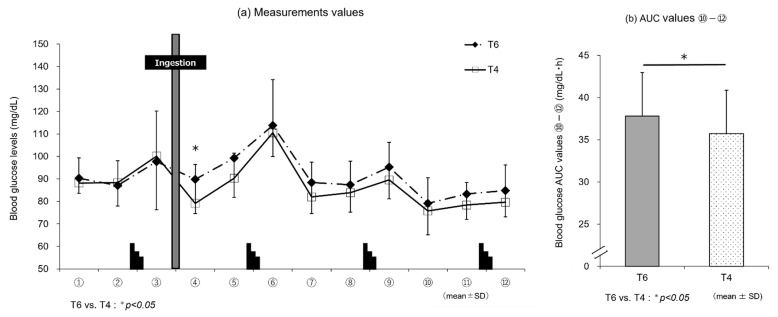
Changes in the blood glucose levels with trial drinks (experiment 2). Comparison between (**a**) measurements and (**b**) area under the curve (AUC) ⑩–⑫ values of trial drinks (*n* = 10). T6 vs. T4: * *p* < 0.05. T6, 6% trehalose; T4, 4% trehalose.

Figure 4a shows the changes in blood glucose levels by trial drink (T6 or T4). The blood glucose levels increased at the end of each set of the Wingate test (time points ③, ⑥, ⑨, and ⑫) with both T6 and T4. At 15 min after drink ingestion (④), the blood glucose levels were significantly higher for T6 (89.8 ± 17.3 mg/dL) than T4 (79.1 ± 10.4 mg/dL) (t[9] = 2.56, p < 0.05, d = 0.81). After that, the levels remained higher in T6 than T4, although no significant differences were observed between these trial drinks. The AUC ⑩–⑫ value in the later stages of the exercise was significantly higher for T6 (37.8 ± 5.3 mg/dL·h) than T4 (35.7 ± 4.7 mg/dL·h) (t[9] = 2.76, *p* < 0.05, *d* = 0.87) (Figure 4b).

### 3.3. Individual Changes in Mean Power Value and Blood Glucose AUC

**Figure 5:**
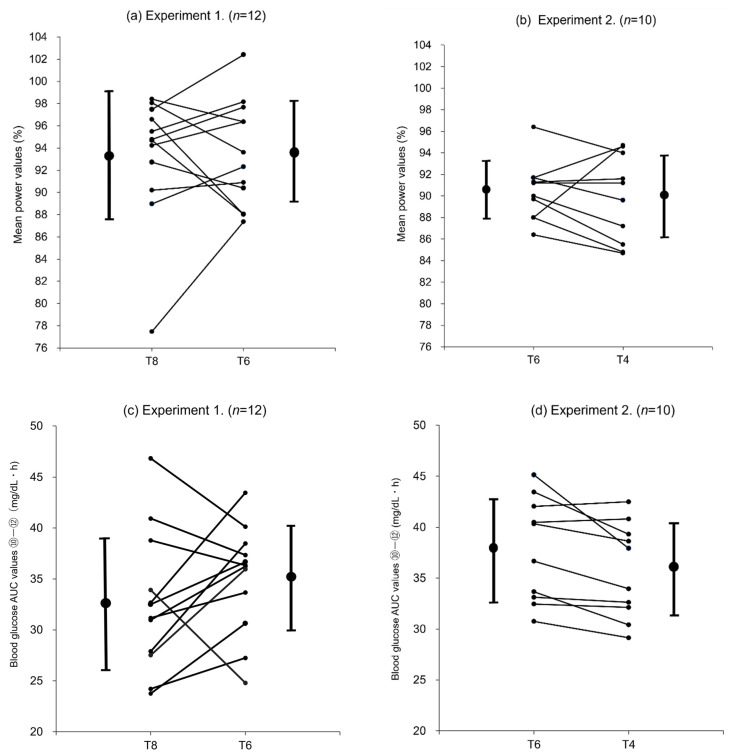
Individual changes in mean power values and blood glucose AUC. (**a**) Experiment 1: Changes in subjects’ mean power values (4th: T8 and T6) (*n* = 12). (**b**) Experiment 2: Changes in subjects’ mean power values (4th: T6 and T4) (*n* = 10). (**c**) Experiment 1: Changes in subjects’ blood glucose (AUC ⑩–⑫: T8 and T6) (*n* = 12). (**d**) Experiment 2: Changes in subjects blood glucose (AUC ⑩–⑫: T6 and T4) (*n* = 10).

Figure 5a,b show the changes in mean power value for the fourth set and Figure 5c,d shows changes in blood glucose AUC ⑩–⑫ between different concentrations of trial drinks by participant separately for experiments 1 (T8 vs. T6) and 2 (T6 vs. T4).

In experiment 1, the mean power values for all participants were comparable with T8 (93.3 ± 5.8%) and T6 (93.5 ± 4.8%) (Table 2). However, at the individual level, the mean power value was higher with T6 than T8 for 7 of the 12 participants (Figure 5a). The mean blood glucose AUC ⑩–⑫ was higher with T6 than T8 (Figure 3b), although some participants experienced changes in a different manner (Figure 5c).

In experiment 2, the mean power values (percent ratios) for all participants were comparable with T6 (90.4 ± 2.8%) and T4 (89.8 ± 4.0%) (Table 3), and the mean blood glucose AUC ⑩–⑫ was significantly higher with T6 than T4 (Figure 4b; *p* < 0.05). However, some participants experienced changes in a different manner (Figure 5b,d).

### 3.4. Respiratory Exchange Ratio (RER)

**Figure 6:**
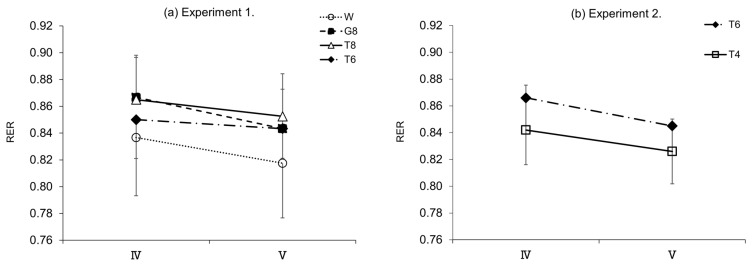
RER assessments. (**a**) Experiment 1 (*n* = 12); (**b**) experiment 2 (*n* = 10). W, water; G8, 8% glucose; T8, 8% trehalose; T6, 6% trehalose; T4, 4% trehalose.

Figure 6 shows the changes in RER in the later stages of the exercise (IV and V) in experiments 1 and 2, which were determined by analyzing the expired gas collected during the second half (15 min) of the constant-load exercise. In experiment 1 (Figure 6a), a significant main effect was found (IV: F[3, 33] = 3.597, *p* < 0.05, $\eta_{2}^{p}$= 0.246, V: F [3, 33] = 3.657, *p* < 0.05, $\eta_{p}^{2}$ = 0.249), although no significant differences in RER were observed between the trial drinks for periods IV and V. In experiment 2 (Figure 6b), no significant differences in RER were noted between the trial drinks for periods IV and V. However, the effect size in period IV was classified as medium (t[9] = 1.99, p = 0.077, d = 0.63), and the RER tended to be higher for T6 than for T4.

### 3.5. Rating of Perceived Exertion (RPE) and Amount of Ad Libitum Water Intake

There were no significant differences in RPE or ad libitum water intake between the trial drinks until the end of the exercise in experiments 1 and 2.

## 4. Discussion

Our previous studies demonstrated that a single ingestion of trehalose solution during exercise has an effect on maintaining performance in the later stages of prolonged exercise compared to glucose solution [22,23]. Recent studies have shown that ingestion of a solution containing multiple types of carbohydrates is ideal for improving exercise performance, taking advantage of their absorption properties [12,13,29]. To prepare a mixed solution containing trehalose, the concentration of carbohydrates needs to be 8% or less to reduce discomfort or stomach upset due to the increased amount (concentration) of carbohydrates.

Dilute carbohydrate solutions (e.g., up to 6% or 60 g/L) ingested during exercise are usually eliminated from the stomach at the same rate as an equal amount of water [30,31,32,33]. In contrast, a solution with a carbohydrate concentration of >8% is eliminated at a markedly slower rate than water [34]. In a study on the absorption of glucose in the small intestine, there was no limitation on the absorption of a dilute glucose solution ingested during exercise, and all ingested glucose could be converted to blood glucose [35]. The rate-limiting factors for carbohydrate absorption are the total amount and the type of carbohydrate [30]. The carbohydrate concentration is a critical determinant of the gastric emptying rate, because a high concentration may be associated with a slower emptying rate, resulting in impaired carbohydrate absorption.

Interestingly, no differences were observed in exercise performance between the 8 and 4% concentrations in our study.

### 4.1. Relationship between Trehalose Concentration and Exercise Performance

Depletion of muscle glycogen stores during prolonged exercise reduces exercise performance. It is well known that ingesting carbohydrates before or during prolonged exercise increases liver and muscle glycogen stores to improve exercise performance [8]. Such a mechanism during exercise may contribute to the addition of an energy source and prevent the development of hypoglycemia [10].

In our previous study on the effect of single carbohydrate ingestion on the maintenance of performance in prolonged exercise such as a marathon [23], ingestion of T8 was demonstrated to successfully maintain performance until the later stages of exercise without reducing exercise efficiency compared with G8. The slow rise in blood glucose levels, which is characteristic of trehalose, and the accompanying low insulin secretion effect led to the preservation of carbohydrates in the body by inhibiting carbohydrate waste, resulting in the maintenance of performance during the later stages of exercise. This is a feature that differs significantly from glucose.

Experiment 1 compared the mean power values (Table 2). In the fourth set, during the later stages of exercise, the mean power value was significantly higher for T8 than G8 (*p* < 0.05); these results are in agreement with those of a previous study [22]. A comparison of exercise performance between T8 and T6 showed no significant differences in performance despite the difference in trehalose intake. Therefore, T6, with a lower trehalose concentration, maintained performance at a level similar to T8.

Based on this finding, the same experiment was performed using T4, with an even lower concentration, in experiment 2 (Table 3). Exercise performance was found to be comparable for T4 and T6.

Overall, a difference in exercise performance was found between G8 and T8, in which the type of carbohydrate was different, although the carbohydrate (energy) intake was the same. It should also be noted that no differences in exercise performance were found between T8 and T6 or between T6 and T4 despite the difference in carbohydrate (energy) intake.

### 4.2. Relationship between Trehalose Concentration, Blood Glucose Levels, and Insulin Secretion

Trehalose is characterized by a delayed glycemic response and an attenuated insulin response compared to other monosaccharides and disaccharides [17,36,37,38]. In a previous study comparing the effects of ingesting T8 and G8 during exercise [23], the increases in blood glucose levels and insulin secretion were more gradual with T8 than G8. These differences were presumed to involve incretin, a gastrointestinal hormone that stimulates insulin secretion from pancreatic β-cells in a blood glucose-dependent manner. The major incretin hormones are gastric inhibitory polypeptide (GIP) and glucagon-like peptide-1 (GLP-1) [39]. GIP is secreted from K cells abundantly in the duodenum and acts directly on pancreatic β cells [40]. Trehalose hardly stimulates K cells during absorption by the gastrointestinal tract [40], which may explain why insulin secretion was lower after trehalose ingestion than glucose ingestion. GIP secretion from K cells is enhanced in response to glucose ingestion [41]. Insulin promotes cellular glucose uptake and increases intracellular glucose metabolism.

In line with the discussion above, the results of blood glucose measurements in experiment 1 (Figure 3a) show that T6, despite the lower carbohydrate intake, induced higher blood glucose levels in the later stages of exercise than T8 or G8. At the last blood sampling time point, ⑫, the blood glucose level was significantly higher with T6 than G8 (*p* < 0.05). Although no insulin measurements were performed in this study, T6 induced lower blood glucose levels than T8 and G8, suggesting that T6 stimulated GIP secretion only modestly, resulting in less insulin secretion. The lack of stimulation of glucose uptake and metabolism by insulin was considered to allow for the preservation of carbohydrates. This may explain why T6, despite the lower amount of trehalose, induced higher blood glucose levels in the later stages of exercise, leading to performance levels similar to those with T8 (Table 2). In experiment 2, the results of blood glucose measurements (Figure 4a) showed that T6, reflecting the intake of trehalose, induced higher blood glucose levels immediately after ingestion until the end of exercise than T4, and the blood glucose AUC (⑩–⑫) (Figure 4b) was significantly higher with T6 than T4 (*p* < 0.05). However, these differences in blood glucose levels did not affect exercise performance. This may be explained as follows: T4, with a lower amount of trehalose, stimulates GIP secretion only modestly and thus induces lower insulin secretion than T6. Therefore, the stimulation of glucose uptake and metabolism by insulin is suppressed, and lipids are efficiently used, as suggested by the results of RER determination (Figure 6b).

### 4.3. Relationship between Trehalose Concentration and Trehalase Activity

The enzyme trehalase is required for the degradation of the disaccharide trehalose into two glucose molecules. Trehalase, which is secreted from the intestinal brush border, is a specific enzyme that hydrolyzes only trehalose [16]. In a study by Oku et al. investigating trehalase activity at rest [42], increased blood glucose and insulin levels were observed in participants with high trehalase activity who ingested 50 g of trehalose. In contrast, little change was noted in blood glucose levels or insulin secretion in participants with low trehalase activity who ingested 30 g of trehalose, because the trehalose was not fully digested or absorbed in the small intestine. Thus, trehalase activity varies among individuals and affects the degradation of trehalose.

In this study, the mean values of all participants were used for each evaluation. However, as shown in the individual changes in blood glucose AUC (⑩–⑫) in experiment 1 (Figure 5c), the changes in AUC values between T8 and T6 did not necessarily reflect the difference in trehalose intake and varied from subject to subject, suggesting that the subject population consisted of individuals with different levels of trehalase activity. Subjects who had higher blood glucose AUC (⑩–⑫) values after ingesting T8 (40 g) than T6 (30 g) were considered to have high trehalase activity. In these subjects, trehalase was secreted according to the trehalose intake (notably, the amount sufficient to degrade 40 g of trehalose), and the ingested trehalose was probably fully degraded and absorbed as glucose. Subjects with higher blood glucose AUC (⑩–⑫) values after ingesting T6 (30 g) than T8 (40 g) were considered to have low trehalase activity. In these subjects, trehalase was not secreted in an amount sufficient to degrade 40 g of trehalose (T8), and the ingested trehalose was probably transported to the large intestine and not fully degraded and absorbed as glucose in the small intestine. However, after the ingestion of 30 g trehalose (T6), the amount of trehalase appropriate for the trehalose intake was considered to be secreted, and the ingested trehalose was probably efficiently degraded and absorbed as glucose. In experiment 2 (Figure 5d), most participants exhibited lower blood glucose AUC (⑩–⑫) values after ingesting T4 (20 g) than T6 (30 g), suggesting that few differences could be attributable to trehalase activity.

Overall, these comparisons of the changes in blood glucose levels show that some participants who received T8 (40 g trehalose) could not degrade the entire amount of ingested trehalose and therefore could not absorb glucose in an amount consistent with the trehalose intake. Moreover, these participants may have experienced a decline in their gastric emptying rate and eventually glucose absorption, resulting in lower glucose levels than participants who received T6 (30 g trehalose) in the later stages of exercise. However, in participants who received T6 or T4, including those with low trehalase activity, most of the ingested trehalose was probably degraded, and the blood glucose levels were maintained based on the absorbed glucose. In particular, in participants receiving T4 (20 g trehalose), the ingested trehalose was presumed to be efficiently digested and absorbed without being influenced by the gastric emptying rate, leading to higher blood glucose levels being maintained in the later stages of exercise.

Given that exercise performance was similar with T8, T6, and T4, the lowest single dose concentration of 4% (T4, 20 g trehalose) was found to maintain high performance during prolonged exercise. Based on this finding and the slower absorption of trehalose, we propose a new carbohydrate supplement solution idea: to develop various mixed carbohydrate solutions utilizing the absorption, insulin secretion, and taste characteristics of each carbohydrate, e.g., glucose, fructose, HBCD, etc.

There are some limitations of this study. We did not investigate individuals’ trehalase profile, trehalose-degrading enzyme activity, or insulin secretion, and we did not show individual differences due to the degradation and absorption of trehalose in the small intestine. In the future, we need to perform trehalose tolerance tests of participants to assess their exercise performance, blood glucose levels, and insulin secretion.

## 5. Application and Future Research

The ingestion of a trehalose solution, which induces a gradual increase in blood glucose levels and slight insulin secretion, before prolonged endurance exercise such as a marathon is considered unlikely to cause a rapid decrease in blood glucose levels, such as insulin shock. In particular, a trehalose concentration of ≤6% may have little impact on an individual’s ability to degrade trehalose, and even 4% is suggested to be useful in a mixed carbohydrate solution. Trehalose is unlikely to cause a decrease in exercise performance and is expected to preserve carbohydrates in the body and maintain performance in the later stages of exercise. It is possible to prepare special tailor-made trehalose drinks by determining the trehalase activity of individual athletes. However, further studies are required to clarify the effects of mixed solutions of trehalose and other carbohydrates in order to achieve this goal.

## 6. Conclusions

In this study, we conclude that no differences were found in performance in the later stages of high-intensity intermittent exercise for 5 h between groups ingesting trehalose solution at concentrations of 8%, 6%, and 4%.

## Figures and Tables

**Table 1:** Characteristics of participants included in experiments 1 and 2.

		Age	Height	Weight	%Fat	$\dot{V}O_{2}$ Peak	Load (Peak)	$40\%\;\dot{V}O_{2}\mathrm{Peak}$	Load $\left(40\%\;\dot{V}O_{2}\mathrm{Peak}\right)$
		(Year)	(cm)	(kg)	(%)	(mL/min)	(Watt)	(mL/min)	(Watt)
**Experiment 1**	Mean	21.3	174.5	68.6	19.4	3211.6	280.7	1284.6	101.1
(*n* = 12)	SD	1.3	6.8	7.4	2.2	564.0	35.8	225.6	14.0
**Experiment 2**	Mean	21.1	175.6	67.7	18.4	3061.5	273.2	1223.6	100.6
(*n* = 10)	SD	0.7	3.3	4.4	2.6	590.7	44.8	236.3	16.0

**Table 2:** Mean power value assessment and statistical analysis results (experiment 1).

	Trial Drinks				Results of Comparisons between Trial Drinks
	W	G8	T8	T6	
3rd	92.1 ± 4.7	94.7 ± 4.6	96.8 ± 4.0	97.9 ± 2.6	W vs. T6 *
4th	86.8 ± 5.8	88.9 ± 7.2	93.3 ± 5.8	93.5 ± 4.8	W vs. T8 *, W vs. T6 *, G8 vs. T8 *

Values are expressed as mean ± SD; n = 12. W, water; G8, 8% glucose; T8, 8% trehalose; T6, 6% trehalose. Comparison of mean power values in Wingate test (3–4 sets) for each trial drink (W, G8, T8, T6). Performance means in each set (3rd and 4th) were compared with 1st mean power value set at 100 for each trial drink. No significant differences in mean power values were observed between T8 and T6 in 3rd or 4th set. * *p* < 0.05.

**Table 3:** Mean power value assessment and statistical analysis results (experiment 2).

	Trial Drinks		Results of Comparisons between Trial Drinks
	T6	T4	
3rd	95.9 ± 2.7	94.6 ± 4.8	n.s.
4th	90.4 ± 2.8	89.8 ± 4.0	n.s.

Values are expressed as mean ± SD; *n* = 10. T6, 6% trehalose; T4, 4% trehalose. Comparison of mean power values in Wingate test (3–4 sets) for each trial drink (T6, T4). Performance means in each set (3rd and 4th) were compared with 1st mean power value set at 100 for each trial drink. No significant differences in mean power values were observed between T6 and T4 in 3rd or 4th set. n.s., not significant.

## Data Availability

The data presented in this study are available on request from the corresponding author and the permission of all parties involved in the study.
